# Correction: Income inequality and its relationship with loneliness prevalence: A cross-sectional study among older adults in the US and 16 European countries

**DOI:** 10.1371/journal.pone.0315729

**Published:** 2024-12-10

**Authors:** Thamara Tapia-Muñoz, Ursula M. Staudinger, Kasim Allel, Andrew Steptoe, Claudia Miranda-Castillo, José T. Medina, Esteban Calvo

An additional affiliation is missing for the seventh author. Esteban Calvo is also affiliated with the Millennium Nucleus for the Evaluation and Analysis of Drug Policies, Santiago, Chile.

The following information is missing from the Funding statement: (EC) Research and Development National Agency–FONDECYT REGULAR-1140107 and Research and Development National Agency–Millennium Science Initiative Program—Millennium Nucleus on Sociomedicine—NCS2021_013 and Millennium Nucleus for the Evaluation and Analysis of Drug Policies—NCS2021_003 awarded to E.C. www.anid.cl www.iniciativamilenio.cl.
