# Supplementary material for: Income inequality and its relationship with loneliness prevalence: A cross-sectional study among older adults in the US and 16 European countries
Source: PLoS One. 2022 Dec 6;17(12):e0274518. doi: 10.1371/journal.pone.0274518 (PMC9725142; doi:10.1371/journal.pone.0274518)
Supplement: S1 File — (DOCX) [file pone.0274518.s001.docx]

**S3 Tables and figures. Predictive models for the prevalence of loneliness**

**Table A.** Individual-level independent variables distribution per country

|  | Age (mean) | Female (%) | Married/partnered (%) | College and above (%) | Workers (%) | Self-perceived Health | Depressive Mood  (%) | Pain prevalence  (%) |
| --- | --- | --- | --- | --- | --- | --- | --- | --- |
| US | 67 | 58 | 34 | 26 | 18 | 3.01 | 35 | 36 |
| Austria | 66 | 55 | 28 | 33 | 28 | 3.03 | 41 | 42 |
| Belgium | 66 | 58 | 30 | 13 | 20 | 2.62 | 44 | 48 |
| Czech Republic | 65 | 53 | 22 | 42 | 42 | 3.50 | 31 | 31 |
| Denmark | 68 | 56 | 28 | 16 | 31 | 3.23 | 10 | 40 |
| England | 68 | 61 | 32 | 22 | 30 | 2.13 | 48 | 50 |
| Sweden | 67 | 57 | 31 | 21 | 23 | 2.79 | 47 | 57 |
| Estonia | 64 | 52 | 20 | 30 | 36 | 2.76 | 46 | 38 |
| France | 67 | 56 | 20 | 34 | 38 | 2.92 | 30 | 43 |
| Germany | 67 | 54 | 21 | 8 | 22 | 2.73 | 40 | 54 |
| Israel | 64 | 53 | 22 | 19 | 25 | 2.96 | 46 | 43 |
| Italy | 66 | 55 | 21 | 28 | 30 | 3.06 | 31 | 30 |
| Luxemburg | 66 | 57 | 25 | 17 | 16 | 2.73 | 38 | 53 |
| Netherlands | 68 | 54 | 20 | 10 | 23 | 2.68 | 35 | 44 |
| Slovenia | 68 | 53 | 22 | 30 | 32 | 3.32 | 32 | 37 |
| Spain | 66 | 54 | 23 | 17 | 38 | 3.31 | 39 | 26 |
| Switzerland | 69 | 59 | 36 | 24 | 35 | 3.13 | 12 | 39 |

**Table B.** Predictors for loneliness prevalence: single logistic regression results.

|  | OR | SE | Z | p-value | 95% CI | |
| --- | --- | --- | --- | --- | --- | --- |
| Age | 1.041 | 0.003 | 15.740 | 0.000 | 1.036 | 1.047 |
| Male ^a^ | 1.573 | 0.050 | 14.370 | 0.000 | 1.479 | 1.673 |
| Divorced or separated ^b^ | 0.865 | 0.049 | -2.540 | 0.011 | 0.774 | 0.968 |
| Widowed ^b^ | 0.563 | 0.033 | -9.900 | 0.000 | 0.503 | 0.631 |
| Single or never married ^b^ | 0.588 | 0.041 | -7.600 | 0.000 | 0.513 | 0.674 |
| GED^c^ | 0.441 | 0.028 | -13.110 | 0.000 | 0.390 | 0.498 |
| High school graduate ^c^ | 3.475 | 0.154 | 28.060 | 0.000 | 3.185 | 3.791 |
| Some college ^c^ | 3.613 | 0.133 | 34.930 | 0.000 | 3.362 | 3.883 |
| College and above ^c^ | 3.505 | 0.200 | 22.030 | 0.000 | 3.135 | 3.919 |
| Out of the labor force^d^ | 2.449 | 0.157 | 14.010 | 0.000 | 2.160 | 2.775 |
| Retired ^d^ | 1.848 | 0.077 | 14.740 | 0.000 | 1.703 | 2.005 |
| Disabled ^d^ | 5.926 | 0.394 | 26.790 | 0.000 | 5.203 | 6.750 |
| Unemployed ^d^ | 3.528 | 0.290 | 15.350 | 0.000 | 3.004 | 4.145 |
| Low limitation ^e^ | 3.036 | 0.136 | 24.760 | 0.000 | 2.780 | 3.315 |
| Moderate limitation ^e^ | 4.644 | 0.255 | 27.920 | 0.000 | 4.169 | 5.172 |
| Severe limitation ^e^ | 5.892 | 0.472 | 22.120 | 0.000 | 5.035 | 6.894 |
| Depressive mood ^f^ | 0.470 | 0.008 | -46.420 | 0.000 | 0.455 | 0.485 |
| Self-percevied Health | 7.045 | 0.257 | 53.490 | 0.000 | 6.559 | 7.568 |
| Pain^g^ | 2.493 | 0.078 | 29.130 | 0.000 | 2.345 | 2.651 |

**Notes**. Logistic regressions. Ref categories. ^a^Women. ^b^Married or partnered. ^c^Less than high school. ^d^Worker. ^e^No limitation. ^f^No depressive mood. ^g^No Pain. * p<0.05 ** p<0.01 ***p<0.001.

**Based on the age differences per country we decided to have a random slope in age.**

**Figure A**. Age-related changes in the prevalence of loneliness by country
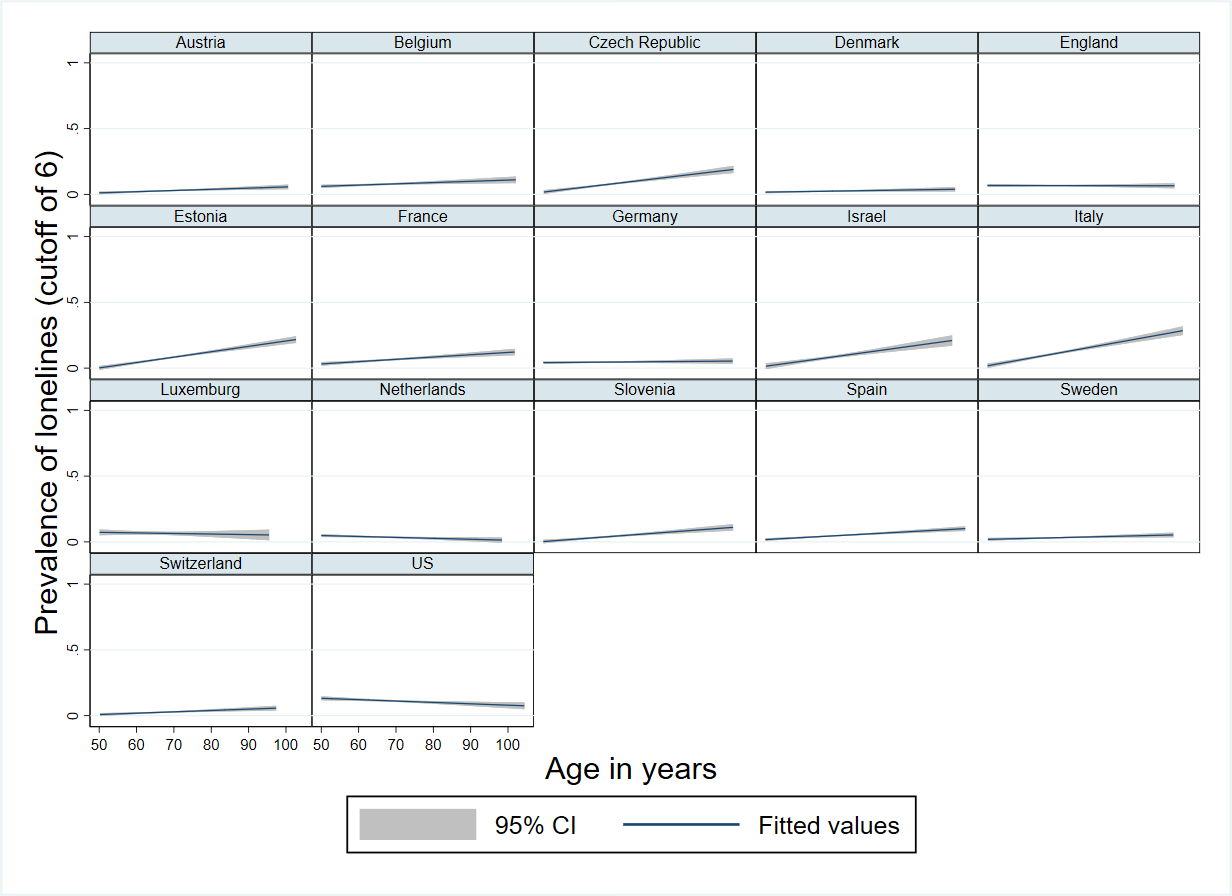


**Note.** Unadjusted prevalence of loneliness (raw values were used).

**Table C:** analysis of US differences in the prevalence of loneliness

| Proportion differences | N | Mean | z | p-value |
| --- | --- | --- | --- | --- |
| US | 7158 | 4.42 (1.63) |  |  |
| Group Mean without US | 78371 | 3.87 (1.38) | 27.6567 | 0.0001 |
| Czech Republic | 5358 | 4.21 (1.42) | 7.6809 | 0.0001 |
| Italy | 4552 | 4.2 (1.66) | 7.0401 | 0.0001 |

**Note.** Conclusion: for country specific estimates use the US as reference category.

**Table D.** Country estimates based on Model 4.

|  | re1^a*^ | re2^b^ | se1^c*^ | se2^d^ | intercept^e^ | slope^f^ |
| --- | --- | --- | --- | --- | --- | --- |
| US | -0.0050 | -0.6498 | 0.0115 | 0.1386 | 4.1408 | 0.0040 |
| Austria | -0.0148 | 0.7751 | 0.0082 | 0.0939 | 2.7159 | -0.0059 |
| Belgium | 0.0220 | 0.4193 | 0.0085 | 0.0907 | 3.0717 | 0.0310 |
| Czech Republic | 0.0155 | -0.3746 | 0.0117 | 0.1529 | 3.8656 | 0.0244 |
| Denmark | -0.0097 | 0.7975 | 0.0074 | 0.0783 | 2.6935 | -0.0007 |
| England | 0.0228 | -0.5735 | 0.0082 | 0.0939 | 4.0645 | 0.0317 |
| Sweden | -0.0013 | -0.1505 | 0.0089 | 0.1078 | 3.6415 | 0.0076 |
| Estonia | 0.0128 | -0.3471 | 0.0095 | 0.1133 | 3.8381 | 0.0217 |
| France | -0.0101 | 0.0302 | 0.0108 | 0.1250 | 3.4608 | -0.0012 |
| Germany | 0.0159 | 0.2247 | 0.0080 | 0.0892 | 3.2663 | 0.0248 |
| Israel | -0.0144 | 0.1570 | 0.0127 | 0.1517 | 3.3340 | -0.0054 |
| Italy | -0.0012 | 0.1376 | 0.0111 | 0.1275 | 3.3534 | 0.0077 |
| Luxemburg | 0.0054 | -0.0468 | 0.0117 | 0.1487 | 3.5378 | 0.0143 |
| Netherlands | -0.0024 | -0.6854 | 0.0087 | 0.1081 | 4.1764 | 0.0065 |
| Slovenia | -0.0038 | 0.0355 | 0.0108 | 0.1258 | 3.4555 | 0.0052 |
| Spain | -0.0157 | -0.4383 | 0.0127 | 0.1547 | 3.9293 | -0.0068 |
| Switzerland | -0.0152 | 0.7415 | 0.0064 | 0.0708 | 2.7495 | -0.0063 |

**Notes.** ^a^ random effect for the country intercept. ^b^ random effect for the country slope. ^c^ standard error for the country intercept. ^d^ standard error for the country slope. ^e^ Slope combined the slope by country, and fixed-effects slope for the whole analytic sample. ^f^ Due the loneliness scale range, the intercept was multiplied per -1 to avoid negative values. *Individual calculation. It displays the first observation per country.

**Figure B.** Age-related changes in the probability of loneliness by country


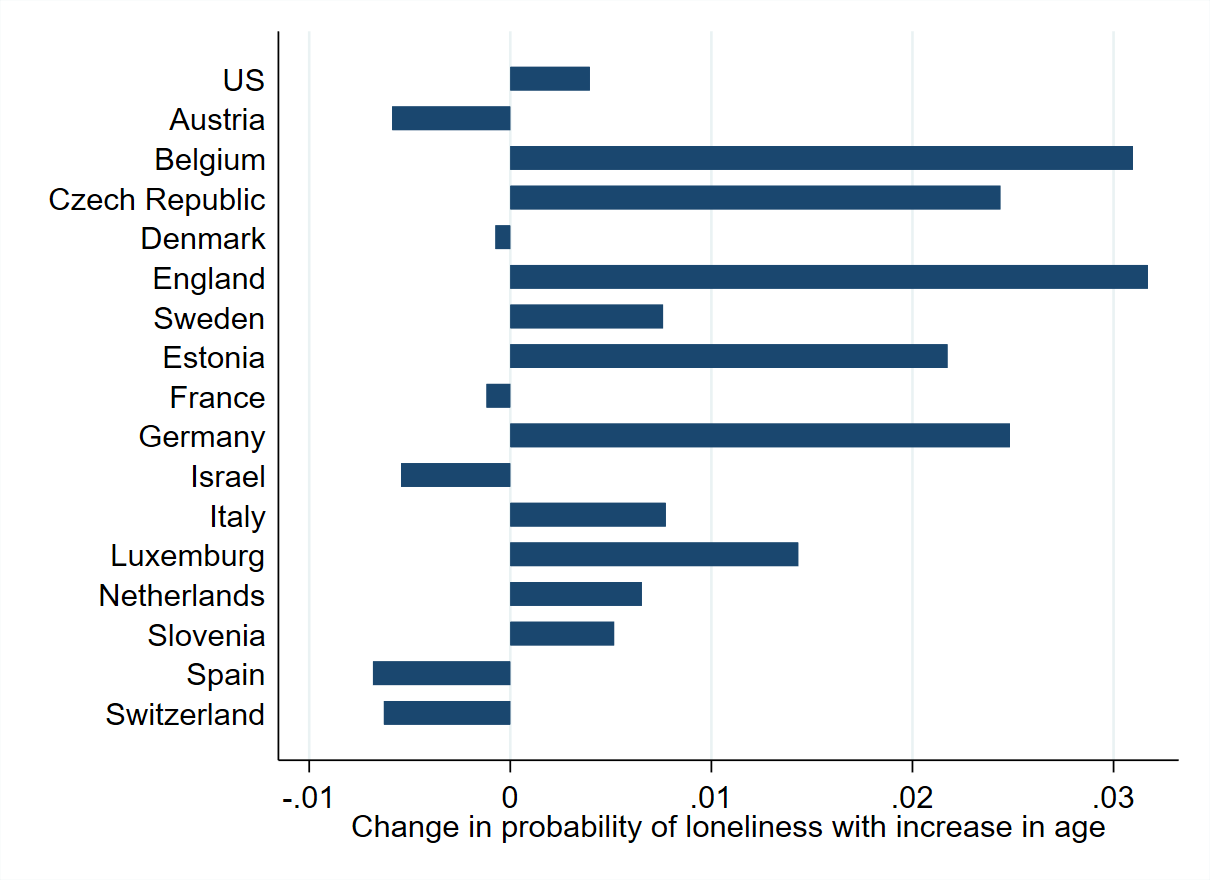


**Note.** Country slope calculated based on Model 4.
