# Supplementary material for: Income inequality and its relationship with loneliness prevalence: A cross-sectional study among older adults in the US and 16 European countries
Source: PLoS One. 2022 Dec 6;17(12):e0274518. doi: 10.1371/journal.pone.0274518 (PMC9725142; doi:10.1371/journal.pone.0274518)
Supplement: S1 Table — (DOCX) [file pone.0274518.s002.docx]

**S1 Table. Analytic sample description**

| Survey | Years | Countries | Total sample size | Analytic sample size |
| --- | --- | --- | --- | --- |
| HRS | 2014 | US | 42,053 | 7,073 |
| ELSA | 2014 | England | 18,523 | 7,934 |
| SHARE | 2013 | 15 European countries | 94,593 | 61,417 |
|  |  | Austria |  | 3,984 |
|  |  | Belgium |  | 5,264 |
|  |  | Czech Republic |  | 5,279 |
|  |  | Denmark |  | 3,891 |
|  |  | Estonia |  | 5,404 |
|  |  | France |  | 4,230 |
|  |  | Germany |  | 5,398 |
|  |  | Israel |  | 2,162 |
|  |  | Italy |  | 4,413 |
|  |  | Luxemburg |  | 1,512 |
|  |  | Netherlands |  | 3,882 |
|  |  | Slovenia |  | 2,807 |
|  |  | Spain |  | 5,968 |
|  |  | Sweden |  | 4,333 |
|  |  | Switzerland |  | 2,980 |
