# Supplementary material for: Income inequality and its relationship with loneliness prevalence: A cross-sectional study among older adults in the US and 16 European countries
Source: PLoS One. 2022 Dec 6;17(12):e0274518. doi: 10.1371/journal.pone.0274518 (PMC9725142; doi:10.1371/journal.pone.0274518)
Supplement: S2 Table — (DOCX) [file pone.0274518.s003.docx]

**S2 Table. Missing data**

94% of the eligible sample had complete cases for loneliness and all the covariates. The missingness did not follow a specific pattern.

**Table A.** Descriptive analysis of missing data

| **Variable** | **Frequency** | **Percentage (%)** |
| --- | --- | --- |
| Loneliness | 613 | 0.78 |
| Age | 4 | 0.01 |
| Gender | 0 | 0 |
| Marital Status | 782 | 0.99 |
| Educational Attainment | 1,766 | 2.24 |
| Labor Force Status | 1,356 | 1.72 |
| Functional limitation | 42 | 0.05 |
| Depressive Mood | 372 | 0.47 |
| Self-reported health | 66 | 0.08 |
| Pain Presence | 100 | 0.13 |

**Table B.** Missing values patterns

| Percent of observations | Functional limitations | Self-perceived health | Pain presence | Depressive mood | Loneliness | Marital Status | Labor force participation | Educational Attainment |
| --- | --- | --- | --- | --- | --- | --- | --- | --- |
| 94% | 1 | 1 | 1 | 1 | 1 | 1 | 1 | 1 |
| 1 missing value | | | | | | | | |
| 2 | 1 | 1 | 1 | 1 | 1 | 1 | 1 | 0 |
| 2 | 1 | 1 | 1 | 1 | 1 | 1 | 0 | 1 |

Note: 1=complete sample
