# Supplementary material for: Income inequality and its relationship with loneliness prevalence: A cross-sectional study among older adults in the US and 16 European countries
Source: PLoS One. 2022 Dec 6;17(12):e0274518. doi: 10.1371/journal.pone.0274518 (PMC9725142; doi:10.1371/journal.pone.0274518)
Supplement: S3 Table — (DOCX) [file pone.0274518.s004.docx]

**Robustness checks. Logistic regression model for loneliness using bootstrap errors with 100 iterations.**

**S4 Table. Observed Bootstrap Normal for model D (N= 75,891)**

|  | OR | SE | Z | p-value | 95% CI | |
| --- | --- | --- | --- | --- | --- | --- |
| Fixed effects |  |  |  |  |  | |
| Intercept | 0.0305 | 0.0098 | -10.8600 | 0.0000 | 0.0162 | 0.0572 |
| Age | 1.0090 | 0.0045 | 1.9800 | 0.0470 | 1.0001 | 1.0179 |
| Male ^a^ | 1.0428 | 0.0481 | 0.9100 | 0.3630 | 0.9526 | 1.1416 |
| Divorced or separated ^b^ | 2.8599 | 0.2500 | 12.0200 | 0.0000 | 2.4095 | 3.3944 |
| Widowed ^b^ | 2.5304 | 0.1528 | 15.3800 | 0.0000 | 2.2480 | 2.8482 |
| Single or never married ^b^ | 3.1674 | 0.1319 | 27.6900 | 0.0000 | 2.9192 | 3.4368 |
| Educational attainment ^c^ | 0.9679 | 0.0414 | -0.7600 | 0.4450 | 0.8901 | 1.0525 |
| Out of the labor force^d^ | 1.4375 | 0.2371 | 2.2000 | 0.0280 | 1.0405 | 1.9860 |
| Retired ^d^ | 1.1211 | 0.2233 | 0.5700 | 0.5660 | 0.7587 | 1.6564 |
| Disabled ^d^ | 1.6820 | 0.3355 | 2.6100 | 0.0090 | 1.1377 | 2.4866 |
| Unemployed ^d^ | 2.0458 | 0.3042 | 4.8100 | 0.0000 | 1.5286 | 2.7379 |
| Low limitation ^e^ | 1.4805 | 0.0722 | 8.0500 | 0.0000 | 1.3456 | 1.6290 |
| Moderate limitation ^e^ | 1.6656 | 0.1138 | 7.4600 | 0.0000 | 1.4567 | 1.9043 |
| Severe limitation ^e^ | 2.1830 | 0.1891 | 9.0100 | 0.0000 | 1.8421 | 2.5869 |
| Depressive mood ^f^ | 4.4024 | 0.5127 | 12.7300 | 0.0000 | 3.5039 | 5.5312 |
| Self-Reported Health | 0.6875 | 0.0144 | -17.9400 | 0.0000 | 0.6600 | 0.7163 |
| Pain ^g^ | 1.2055 | 0.0539 | 4.1800 | 0.0000 | 1.1043 | 1.3160 |
| GINI | 1.4395 | 0.1853 | 2.8300 | 0.0050 | 1.1185 | 1.8527 |
| Random effects |  | Coef. | SE | 95% CI | |  |
| Slope (age) |  | 0.0163 | 0.0163 | 0.0163 | 0.0163 |  |
| Constant |  | 0.4857 | 0.4857 | 0.4857 | 0.4857 |  |
| AIC |  | 28165.84 |  |  |  |  |
| BIC |  | 28248.98 |  |  |  |  |
| Pseudo R^2^ |  | 0.269 |  |  |  |  |

**Notes**. Ref categories. ^a^Men. ^b^Married or partnered. ^c^College and above. ^d^Worker. ^e^No limitation. ^f^No depressive mood. ^g^No Pain.

* p<0.05 ** p<0.01 ***p<0.001. Countries observations were from 1,507 to 6,919 (mean=4,363.2)
