# Supplementary material for: Income inequality and its relationship with loneliness prevalence: A cross-sectional study among older adults in the US and 16 European countries
Source: PLoS One. 2022 Dec 6;17(12):e0274518. doi: 10.1371/journal.pone.0274518 (PMC9725142; doi:10.1371/journal.pone.0274518)
Supplement: S4 Table — (DOCX) [file pone.0274518.s005.docx]

**S5 Table. R-UCLA reliability analysis**

**Table A.** Reliability analysis of the three items from the R-UCLA scale

| Items (all surveys) | Obs. | item-test correlation | average interitem covariance | alpha |
| --- | --- | --- | --- | --- |
| Isolation | 78693 | 0.836 | 0.165 | 0.655 |
| Lack of companionship | 78876 | 0.825 | 0.171 | 0.776 |
| Left out | 78629 | 0.841 | 0.157 | 0.656 |
| Average reliability (all surveys) |  |  | 0.164 | 0.772 |
| HRS |  |  | 0.241 | 0.814 |
| ELSA |  |  | 0.194 | 0.826 |
| SHARE |  |  | 0.147 | 0.752 |

**Notes**: Obs stands for observations.
