# Supplementary material for: Income inequality and its relationship with loneliness prevalence: A cross-sectional study among older adults in the US and 16 European countries
Source: PLoS One. 2022 Dec 6;17(12):e0274518. doi: 10.1371/journal.pone.0274518 (PMC9725142; doi:10.1371/journal.pone.0274518)
Supplement: Attachment — Submitted filename: Reviewer report.docx [file pone.0274518.s006.docx]

**Reviewer’s report:**

This is a referee report on the paper “Income inequality and its relationship with loneliness prevalence: A cross-sectional study among older adults in the US and 16 European countries”.

The authors tried to show a significant association of country-level index of inequality represented by GINI on individual level loneliness by using a multilevel logistic regression model. However, it is uncertain whether those analyses support their conclusion.

Major comments

Is it appropriate to apply multilevel analysis on the data from 17 countries? The reviewer understands the prevalence of outcome significantly varies across the countries however N=17 is too few for multilevel analysis.

In the main result section (3.2 in the text on pages 12-13), the interpretation of the analyses is uncertain.

-In line 217, the authors mentioned “Country differences accounted for seven percent of the total variation of the being lonely.” It is unclear what is the number “7%”. Also, the next sentence “There was statistically significant variability in the odds of loneliness between the countries” is not understandable based on the OR of constant value in the fixed effect. It is not clear what beta01 means (beta01 is not defined in the equation in the method section).

Please, unify an expression of terms. For instance, the authors uses Self reported health and self-perceived health. This mixed expression is confusing readers.

Table B in S2 is not easy to understand.

Minor comments

In line 77 on page 5, R-UCLA suddenly appears. Please, explain what this means at the first use in the text even if the authors explained it in detail after this section.

In Fig. 1, in the column of ELSA, the number of bottoms is “N=7934”. Why does the number of participants increase after incomplete cases are excluded?

In line 177 on page 9, the authors mentioned: “ see Table B in S3 Tables”. Please, mention a result in the result section.

In Table 1, values of frequency and mean are mixed. Unit of age is not shown and the value of Self-Reported Health is unclear. These make the table difficult to understand.

The numbering of the tables is confusing. There are Table A and B in S2 and S3 Tables. The authors may change to for example “S2-A”.
